# EARLY DETECTION AND MANAGEMENT OF COGNITIVE IMPAIRMENT PRACTICES AMONG PRIMARY CARE PROVIDERS

**DOI:** 10.1093/geroni/igad104.1948

**Published:** 2023-12-21

**Authors:** John Omura, Roshni Patel, Benjamin Olivari, Lisa McGuire

**Affiliations:** Centers for Disease Control and Prevention, Atlanta, Georgia, United States; Cyberdata Technologies (CDC Contractor), Atlanta, Georgia, United States; CDC, Atlanta, Georgia, United States; CDC, Atlanta, Georgia, United States

## Abstract

Primary care providers (PCPs) play a critical role in the early detection and management of cognitive impairment and dementia. Understanding their current practice patterns can help identify potential areas of need for additional support. Using 2022 DocStyles survey data (n=1,182), this study assessed US PCPs’ confidence levels in identifying symptoms of cognitive impairment and the actions they take when identifying a patient with cognitive impairment. In terms of their ability to identify symptoms of cognitive impairment, 22.3% of PCPs reported being very confident, 53.2% somewhat confident, 18.3% slightly confident, and 6.2% not at all confident or did not know. PCPs reported taking a variety of actions when they identify a patient with symptoms of cognitive impairment: 70.1% assess for potential underlying causes, 69.4% review medications, 65.7% conduct a cognitive assessment or test, 59.7% refer to a neurologist or other specialist, 43.3% coordinate with a care team, and 20.9% refer to community resources. Compared to PCPs who were very or somewhat confident, those who were slightly or not at all confident were less likely to report taking each action (e.g., 35.1% vs. 75.4% conduct a cognitive assessment or test, respectively), except for being more likely to refer to a neurologist or other specialist (68.8% vs. 56.9%) (p< 0.001). PCP practices in early detection and management of cognitive impairment vary by their confidence level. Findings can inform the development of resources to help increase PCPs’ confidence and support their practices in early detection and management of cognitive impairment and dementia.

